# Absence of Stressful Conditions Accelerates Dexterous Skill Acquisition in Surgery

**DOI:** 10.1038/s41598-019-38727-z

**Published:** 2019-02-11

**Authors:** Ioannis Pavlidis, Dmitry Zavlin, Ashik R. Khatri, Amanveer Wesley, George Panagopoulos, Anthony Echo

**Affiliations:** 1 Computational Physiology Laboratory, University of Houston, Houston, Texas USA; 2 Institute of Reconstructive Surgery, Houston Methodist Hospital, Houston, Texas USA

## Abstract

The negative impact of strong sympathetic arousal on dexterous performance during formal surgical training is well-known. This study investigates how this relationship might change if surgical training takes place as a hobby in an informal environment. Fifteen medical students volunteered in a 5-week training regimen and weekly performed two standardized microsurgical tasks: circular cutting and simple interrupted suturing. Time was taken and two independent reviewers evaluated the surgical proficiency. The State Trait Anxiety Inventory (STAI) and the NASA Task Load Index (NASA-TLX) questionnaires measured subjective anxiety and workload, respectively. A high-resolution thermal imaging camera recorded facial imagery, from which a computational algorithm extracted the perinasal perspiration signal as indicator of sympathetic arousal. Anxiety scores on STAI questionnaires were indifferent for all five sessions. The continuously measured arousal signal from the thermal facial imagery was moderate and did not correlate with surgical proficiency or speed. Progressive experience was the strongest contributor to improved skill and speed, which were attained in record time. It appears that dexterous skill acquisition is facilitated by the absence of strong arousals, which can be naturally eliminated in the context of informal education. Given the low cost and availability of surgical simulators, this result opens the way for re-thinking the current practices in surgical training and beyond.

## Introduction

Pavlidis *et al*.^1^ demonstrated that laparoscopic training in a surgery school is associated with strong arousals, manifesting stressful conditions. These arousals precipitate fight-or-flight responses, the net effect of which are fast, mindless actions leading to errors, and the sustenance of a vicious cycle. The researchers found no performance improvement in untrained surgery residents, after five practice sessions - a sign that high stress levels impede dexterous skill acquisition.

The source of strong arousals during surgical training is an open question. We are aware of no study that separated stressors inherent to surgical tasks from environmental stressors. Hence, while the negative effect of stress on surgical training is well documented^1–3^, the question of what happens if environmental stress factors are managed remains underexplored. Here we investigate how the absence of stressful environmental conditions during microsurgical training affects arousal levels, proficiency, and speed. We remove stressful environmental factors, leaving only the inherent challenge of the surgical tasks, by changing the educational model. Instead of recruiting subjects among surgical interns that undergo formal inanimate training, we recruit lowerclassmen medical students with interest in surgery. Furthermore, we carry out unsupervised training in a relaxed atmosphere outside the medical school. Hence, the participants do not have any stakes or pressures - their only motivation is their love for surgery, and they view the training course as a hobby.

We chose inanimate training in microsurgery as the experimental testbed, because it is challenging, easy to administer in any environment, and has broad applications. Indeed, the field of microsurgery is a subspecialty across several surgical disciplines but plays a particularly large role in plastic and reconstructive surgery. The common ground of all microsurgical procedures is the use of magnifying devices, such as loupes. Microsurgical cases typically entail the preparation of small vessels, nerves, or other vital tissue and their reanastomosis with other anatomical structures^4^. The primary goal is to return amputated tissue to the body and achieve wound closure^5^.

**Figure 1:**
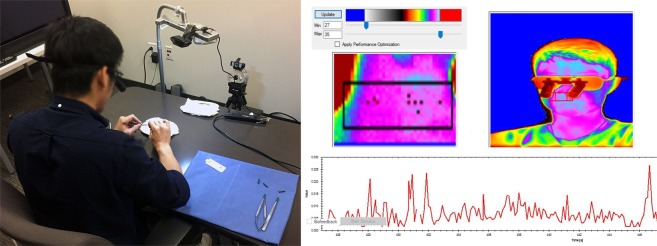
Experimental setting and measurements. (Left) Weekly microsurgical setup. (Right) Facial thermal imagery with region of interest from where the perinasal perspiration signal is extracted.

**Figure 2:**
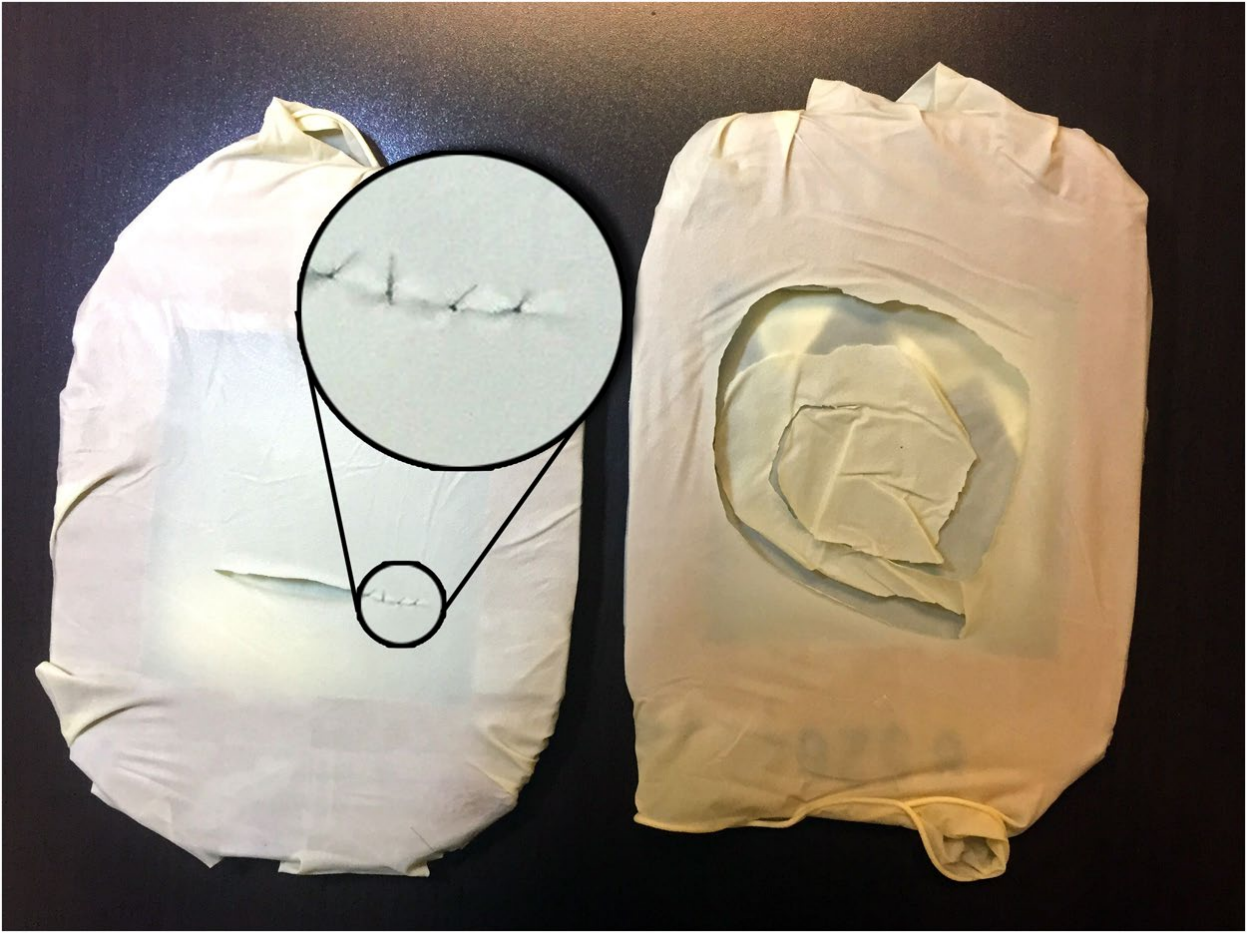
Samples of the 5 × 5 cm rubber models and completed tasks; sutures were magnified for better visibility. On the left is the suturing model and on the right is the cutting model, after the completion of a subject trial.

Inanimate microsurgical training can take place at any desktop using mobile instruments and affordable materials (Figs 1 and 2); thus, it is uniquely suited to ubiquitous courses on surgical skill. Furthermore, because such training does away with the traditional ‘Halstedian’ model^6^ associated with operating room (OR) apprenticeship, conforms with general trends in surgical education. Indeed, microsurgical tasks on an actual patient can be very stressful and technically challenging^7^. Small and refined movements are vital in order not to damage any anatomical entity. Working with minute anatomical structures would put a surgical novice at the unfavorable end of Yerkes-Dodson’s stress-performance chart^8^, with safety implications for the patient under his/her care. For this reason, in microsurgery, but also in laparoscopic surgery, training has moved towards inanimate modules^7,9–12^. The underlying objective of such modules is to educate residents in a way that will increase rather than compromise patient safety when they assume duties in the OR^13,14^.

## Methods

### Subjects

The student surgical societies of three allopathic medical schools in Houston, Texas were contacted for voluntary study participants. The only exclusion criterion was prior experience in microsurgery. After a brief instruction presentation (week 0) by the senior author (A.E.), the subjects weekly performed two standardized microsurgical tasks in our computational lab (weeks 1–5). At each of the five sessions, continuous thermal imaging was used to monitor the subjects’ perinasal perspiration^15^ at baseline and during each of the two microsurgical tasks (Fig. 1). Perinasal perspiration is a peripheral indicator of sympathetic responses^15^. Questionnaires taken in each session evaluated the students’ preoperative vs. postoperative anxiety, as well as their workload perceptions. The institutional review boards (IRB) of both the Methodist Hospital and the University of Houston approved this research. All participating students provided written informed consent, and the study was carried out according to the relevant rules and regulations. The subject appearing in Fig. 1 provided additional informed consent for the publication of his identifying image in an online open-access publication.

### Baseline and Microsurgical Tasks

Each session started with a baseline, where the subjects sat quietly in a chair for 5 min. The mean perinasal perspiration during the baseline of each session *i*, for each subject *k* was used as his/her sympathetic tonic level for the day, and was subtracted from his mean perinasal perspiration (*PP*) in the two microsurgical tasks of session *i*. The thus formed ${\rm{\Delta }}(\overline{PP})$ variables were comparable across subjects because they normalized inter-individual tonic variability. Following the baseline, the subjects performed two microsurgical tasks, cutting and suturing, in a randomized order. To execute these tasks the subjects used 4.0x loupe magnification, microsurgical instruments, and 8-0 Ethicon® (Ethicon Inc., Somerville, New Jersey) microsurgical suturing material. For the cutting task, the subjects had to cut a circle within a 5 × 5 cm rubber model and then continue cutting inwards for another half lap (total of 540°). This task had a time limit of 10 min and would be considered incomplete if the subjects failed to finish the 540° loop. For the suturing task, the subjects first made a linear 3 cm cut in a separate 5 × 5 cm rubber model and then proceeded to place 6 single interrupted stitches to close the defect (Fig. 2). The time limit was set at 20 min. Ideally, the subjects had to place all 6 stitches during these 20 min or as many as they could until the clock ran out. Our senior author (A.E.) has previously studied such rubber models in microsurgical training modules^7^.

Two independent reviewers assessed the subjects’ microsurgical proficiency following a global scale^16^; the lowest possible score was 6 and the highest possible score was 30 points. The time taken to finish a task (or the time limit if the subject ran out of time) and the number of stitches placed during the suturing task were recorded. From these primary variables we derived the mean subtask time, which is a measure of surgical speed with superior analytic properties^1^. The cutting task consisted of a single subtask and thus, the mean subtask time coincided with the task time in that case. For the suturing task, the mean subtask time was obtained by dividing the total time spent on the task by the number of stitches completed.

### Questionnaires

Upon the completion of the consenting process, we collected the students’ demographic details using an ad hoc form. Furthermore, we evaluated the anxiety status and the subjective postoperative workload impression using validated questionnaires. The trait module (TAI) of the State-Trait Anxiety Inventory (STAI)^17^ was administered to the subjects once at the start of the study; the state module (SAI) was administered at the beginning and at the end of each session. STAI consists of 20 statements where the subjects are asked to agree or disagree on 1–4 Likert scales. The total score is between 20 and 80 points; higher values indicate greater anxiety. The NASA Task Load Index (NASA-TLX)^18^ was administered at the end of each task in each training session. More precisely, we used the short version of the questionnaire without sub-weightings of the scales since it has similarly high validity with the longer version^19^. This multidimensional instrument consists of six Likert subscales (Mental, Physical, Temporal, Performance, Effort, Frustration), with each subscale scored between 1 and 20 points. The NASA-TLX measures a participant’s subjective assessment of the workload. Higher scores suggest a greater demand or burden associated with the assigned work.

### Thermal Imaging

We imaged thermally the subjects’ faces during the baseline, cutting, and suturing tasks in each session. For that purpose we used a Tau 640 long-wave infrared (LWIR) camera (FLIR Systems, Wilsonville, OR); it features a small size (44 × 44 × 30 mm) and adequate thermal (50° mK) and spatial resolution (640 × 512 pixels). The Tau 640 camera was outfitted with a LWIR 35 mm lens f/1.2. Thermal data were collected at a frame rate of 7.5 fps. A computational algorithm^15^ was extracting a measure of instantaneous perspiration by operating upon tissue bounded by the upper lip and the nostrils (i.e., upper orbicularis oris region) - Fig. 1. Perinasal perspiration is an alternative to palmar electrodermal activity (EDA), and thus a valid measure of sympathetic arousal^1^. To ensure that the perinasal perspiration algorithm operated on the perinasal area despite head motions, we used a tracker^20^ to follow the region of interest around the image plane. The extracted perinasal perspiration signal was filtered via a Fast Fourier Transform (FFT).

### Statistics

For all statistical analyses of the data we used R Version 3.4.4 (The R Foundation for Statistical Computing, Vienna, Austria). We did hypothesis testing, setting levels of significance at *α* = 0.05 designated by *, *α* = 0.01 designated by **, or *α* = 0.001 designated by ***.

### Code availability

The R code used for the data analysis is available at the Open Science Framework: https://osf.io/9he58/.

## Results

We designed a prospective observational cohort study using young medical students as novice microsurgical trainees; the testbed was inanimate. We had two key goals: First, to investigate if the proficiency and speed of these students could improve over a short-term unsupervised practice course in a controlled, but stress-free environment. Second, to examine whether sympathetic arousal, which was measured thermophysiologically and psychometrically, negatively impacted the students’ microsurgical efficiency under the said conditions. The sympathetic validity of the thermophysiological channel was previously confirmed in a formal residency training program on laparoscopic surgical tasks^1^.

### Subject characteristics

Of the 21 medical students that originally signed consent forms and began the study, only 15 (10 male, 5 female) finished all five sessions of the experiment and are included in this article. Table 1 displays their demographic and personal details in the form of descriptive statistics. The sample’s age statistic was 23.1 ± 1.3 years and the great majority of the subjects (73.3%) were first year students. As per the exclusion criteria, no subject had prior experience in microsurgery. During the 5-week training, the subjects significantly (*p* < 0.001) strengthened their perception of surgical skills from 1.1 ± 0.4 to 3.0 ± 1.0 points on a 1–5 Likert scale (Table 1).

**Table 1:** Subjects’ personal characteristics.

Variable	Subjects
Gender	
Male	*n* = 10 (66.7%)
Female	*n* = 5 (33.3%)
Age	*n* = 15
Years (mean ± SD)	(23.1 ± 1.3)
Year in Medical School	
Year 1	*n* = 11 (73.3%)
Year 2	*n* = 2 (13.3%)
Year 3	*n* = 2 (13.3%)
Prior surgical rotations	*n* = 2 (13.3%)
Prior microsurgical courses	*n* = 0 (0.0%)
Session 1: perceived surgical skill (mean ± SD)	*n* = 15 (1.1 ± 0.4)^†^
Session 5: perceived surgical skill (mean ± SD)	*n* = 15 (3.0 ± 1.0)^†^

^†^Scale: 1 (very poor) to 5 (very good), *p* < 0.001.

The scores for the trait module of the STAI questionnaire were normal (38.8 ± 7.5 points on a 20–80 scale). The mean scores for the preoperative|postoperative state modules ranged between 30.2 | 34.5 and 31.6 | 37.6. Therefore, the subjects exhibited stable, normal anxiety levels.

### Factor analysis for surgical proficiency and speed

We constructed generalized linear models with mixed effects aiming to capture the statistical relationship between certain independent variables and the proficiency scores the subjects received (Tables 2 and 3), as well as the speed with which they completed subtasks (Tables 4 and 5). In all these models, the subjects act as random effects, symbolized by 1|*S* in Eqs 1–4.

**Table 2:** Results of the generalized linear model for the outcome measure surgical proficiency, when independent variables include physiological indicators - Eq. 1.

Variable	Estimate	Std. Error	*t* value	*p* value
Session 2^a^	4.072	0.520	7.829	0.000***
Session 3^a^	6.114	0.511	11.968	0.000***
Session 4^a^	7.474	0.541	13.825	0.000***
Session 5^a^	7.415	0.518	14.310	0.000***
Task Suturing^b^	−1.261	0.334	−3.780	0.000***
$\mathrm{ln}({\rm{\Delta }}\overline{PP})$	−0.146	0.281	−0.517	0.606
Scorer No 2^c^	0.204	0.332	0.616	0.539

Repeat training sessions and the type of task emerge as the only predictors.

^a^*Compared to Session 1*.

^b^*Compared to Cutting Task*.

^c^*Compared to Scorer No 1*.

**Table 3:** Results of the generalized linear model for the outcome measure surgical proficiency, when independent variables include psychometric indicators - Eq. 2.

Variable	Estimate	Std. Error	*t* value	*p* value
Session 2^a^	2.903	0.560	5.179	0.000***
Session 3^a^	5.069	0.550	9.219	0.000***
Session 4^a^	7.036	0.573	12.277	0.000***
Session 5^a^	6.529	0.579	11.275	0.000***
Task Suturing^b^	−1.314	0.578	−2.274	0.024*
SAI	−0.032	0.026	−1.244	0.215
TLX-Mental	−0.024	0.095	−0.251	0.802
TLX-Physical	0.223	0.086	2.593	0.010*
TLX-Temporal	0.036	0.059	0.613	0.540
TLX-Performance	−0.119	0.044	−2.707	0.007**
TLX-Effort	0.029	0.077	0.376	0.708
TLX-Frustration	−0.240	0.066	−3.653	0.000***
Scorer No 2^c^	0.296	0.315	0.938	0.349

Repeat training sessions and the type of task emerge as the key predictors.

^a^*Compared to Session 1*.

^b^*Compared to Cutting Task*.

^c^*Compared to Scorer No 1*.

**Table 4:** Results of the generalized linear model for the outcome measure mean subtask time (aka surgical speed), when independent variables include physiological indicators - Eq. 3.

Variable	Estimate	Std. Error	*t* value	*p* value
Session 2^a^	−279.614	58.022	−4.819	0.000***
Session 3^a^	−335.201	57.045	−5.876	0.000***
Session 4^a^	−386.965	60.024	−6.447	0.000***
Session 5^a^	−398.376	57.754	−6.898	0.000***
Task Suturing^b^	125.704	37.231	3.376	0.001**
$\mathrm{ln}({\rm{\Delta }}\overline{PP})$	−0.994	30.171	−0.033	0.974

Repeat training sessions and the type of task emerge as the only predictors.

^a^*Compared to Session 1*.

^b^*Compared to Cutting Task*.

**Table 5:** Results of the generalized linear model for the outcome measure mean subtask time (aka surgical speed), when independent variables include psychometric indicators - Eq. 4.

Variable	Estimate	Std. Error	*t* value	*p* value
Session 2	−168.854	59.242	−2.850	0.005**
Session 3	−236.705	58.276	−4.062	0.000***
Session 4	−320.037	59.784	−5.353	0.000***
Session 5	−304.365	60.173	−5.058	0.000***
Task Suturing	72.367	60.343	1.199	0.233
SAI	0.331	2.538	0.130	0.896
TLX-Mental	−5.286	8.488	−0.623	0.535
TLX-Physical	−2.927	7.802	−0.375	0.708
TLX-Temporal	−12.582	5.774	−2.179	0.031*
TLX-Performance	5.781	4.166	1.388	0.168
TLX-Effort	7.106	7.639	0.930	0.354
TLX-Frustration	24.127	6.572	3.671	0.000***

Repeat training sessions emerge as the key predictors.

^a^*Compared to Session 1*.

^b^*Compared to Cutting Task*.

Model #1 (Eq. 1) features a physiological predictor, which is the difference of the mean perinasal perspiration between the subject’s task signal and his/her resting baseline, in the context of a training session; the predictor is corrected logarithmically due to its non-linearity: $\mathrm{ln}({\rm{\Delta }}\overline{PP})$. This model revealed significant proficiency score improvement as the subjects progressed with their five training sessions (*p* < 0.001 with positive factor estimates in all sessions - Table 2). Performing the suturing task had a significantly negative impact on the score (*p* < 0.001 with negative factor estimate), which is consistent with the well-known difficulty of this task. Interestingly, the sympathetic arousal levels calculated via the normalized perinasal perspiration $\mathrm{ln}({\rm{\Delta }}\overline{PP})$ were moderate and unrelated to the proficiency scores (*p* > 0.05 - Table 2), thanks likely to the informal educational framework of the study design. The proficiency scores were not affected by the reviewer judging the students (*p* > 0.05 - Table 2), which suggests inter-rater agreement.

Physiological Model #1

1 $$Score\sim 1+Session+Task+\,\mathrm{ln}({\rm{\Delta }}\overline{PP})+Scorer+1|S$$

In Model #2 (Eq. 2), we replaced the physiological variable with the psychometric measurements. These measurements included the anxiety induced by the tasks, as recorded by the scores of the postoperative vs. the preoperative State Anxiety Inventory (SAI) in each session. They also included the six subscales of the NASA-TLX instrument, measuring workload perceptions in each session: ${\rm{Mental}}\equiv  {\mathcal M}$; ${\rm{Physical}}\equiv {\mathscr{P}}$; ${\rm{Temporal}}\equiv {\mathscr{T}}$; ${\rm{Performance}}\equiv {P}_{e}$; ${\rm{Effort}}\equiv  {\mathcal E}$; ${\rm{Frustration}}\equiv  {\mathcal F}$. The length of training remained the most important predictor of increased proficiency scores (*p* < 0.001 with positive factor estimates in all sessions - Table 3). Psychometrically measured anxiety (*SAI* in Eq. 2) was not a factor in proficiency, much like physiologically measured arousal was not a factor per Model #1. Subjects who were content with their performance (*p* < 0.01 with negative estimate in the Performance subscale - Table 3) or irritated with the tasks (*p* < 0.001 with negative estimate in the Frustration subscale - Table 3) achieved lower proficiency scores. Higher perceived physical demand (*p* = 0.01 with positive estimate in the Physical subscale - Table 3) correlated with better proficiency scores. Inter-rater agreement was confirmed once again (*p* > 0.05 - Table 3). The consistency of the results between Model #1 and Model #2 confirm the soundness of the study’s design and methods.

Psychometric Model #2

2 $$Score\sim 1+Session+Task+SAI+ {\mathcal M} +{\mathscr{P}}+{\mathscr{T}}+{P}_{e}+ {\mathcal E} + {\mathcal F} +Scorer+1|S$$

We observed similar trends when we replaced proficiency score with mean time per subtask (i.e., speed) as the dependent variable in the generalized linear models. In the thus formed Physiological Model #3 (Eq. 3), the strongest predictor of decreased subtask time (thus, higher surgical speed) was the number of microsurgical training sessions (*p* < 0.001 with negative factor estimates in all sessions - Table 4). Suturing subtasks were correlating with larger mean times (*p* = 0.001). Arousal levels, as expressed by the perinasal perspiration channel, did not correlate with mean subtask time (*p* > 0.05), much like in the case of proficiency scores.

Physiological Model #3

3 $$Time\sim 1+Session+Task+\,\mathrm{ln}({\rm{\Delta }}\overline{PP})+1|S$$

In Model #4 (Eq. 4), we replaced the physiological predictor of Model #3 with the psychometric predictors. This is part of our approach to predict surgical performance (proficiency score or mean subtask time) through either a physiological or psychometric channel. As in the Physiological Model #3, the strongest predictor of decreased subtask time was the number of microsurgical training sessions (*p* < 0.001 with negative factor estimates in all sessions - Table 5). Psychometrically measured anxiety was not a factor in time performance, much like physiologically measured arousal was not a factor in Model #3. Subjects who felt urgency featured reduced subtask times (*p* < 0.05 with negative estimate in the Temporal subscale). Subjects who were irritated ended up with increased subtask times (*p* < 0.001 with positive estimate in the Frustration subscale - Table 5).

Psychometric Model #4

4 $$Time\sim 1+Session+Task+SAI+ {\mathcal M} +{\mathscr{P}}+{\mathscr{T}}+{P}_{e}+ {\mathcal E} + {\mathcal F} +1|S$$

## Discussion

To investigate if non-stressful conditions affect the relationship between sympathetic arousal and dexterous performance in surgical training, we developed a 5-week informal training regimen where subjects had to perform two standardized microsurgical tasks in randomized order. The tasks were cutting and suturing. Inexperienced allopathic students interested in surgical careers were contacted for voluntary participation in our educational study, which had no repercussion in case of subpar performance.

**Figure 3:**
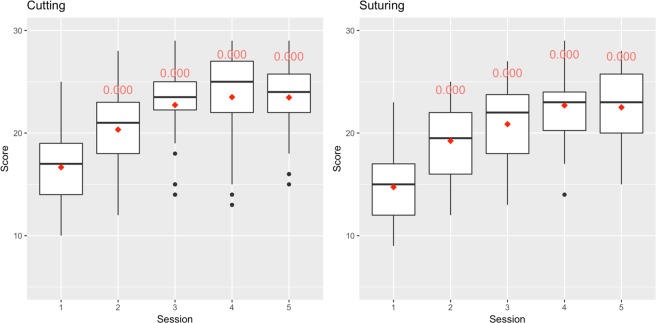
Proficiency score distributions for cutting and suturing, stratified by session number. The numbers in red are the *p* values of the tests against the base session (Session 1) carried out per Eq. 1.

**Figure 4:**
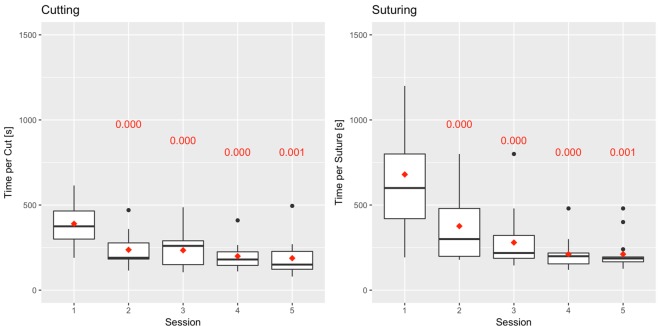
Mean subtask time distributions for cutting and suturing, stratified by session number. The numbers in red are the *p* values of the tests against the base session (Session 1) carried out per Eq. 3.

This informal microsurgical training module yielded strong educational results, with subjects (*n* = 15) exhibiting remarkable improvement in surgical proficiency and speed during the 5-week practice period (Figs 3 and 4). The physiologically measured distress in the form of sympathetic arousal, ${\rm{\Delta }}(\overline{PP})$, was moderate and unchanged throughout the five training sessions; it was not a factor in proficiency performance. The psychometrically measured distress in the form of SAI scores, was in agreement with the physiological indicator. When we replaced proficiency score with mean subtask time (i.e., speed of execution) as the measured outcome, we were able to replicate nearly all statistical associations in the respective models.

In contradistinction to the moderate stress levels and rapid learning results in the present study, previous studies^1–3^ reported high stress levels in surgical trainees and slow learning processes. The main factor that sets the current study apart from these other studies is the educational context. In prior studies, researchers shadowed surgical residents during their mandatory training. In this study, young surgery enthusiasts rather than novice surgeons took up surgical training as hobby.

Hence, it appears that by removing exogenous stress factors, associated with the notoriously competitive and harsh lifestyle of surgery residencies, stress levels during inanimate surgical training plummet. The inherently challenging nature of standardized surgical tasks does not produce overwhelming arousal responses as a stand-alone factor. The residual arousal levels of moderate intensity allow dexterous skill acquisition to flourish.

What is of immense interest is the likely mechanism though which surgical training accelerates under relaxed environmental conditions. As it is reported by Pavlidis *et al*.^1^, high stress levels during formal surgical training precipitate fight-or-flight responses, which manifest as fast action (i.e., high speed). In the absence of experience, these fast actions contribute to high error rates (thus, low proficiency scores), spawning a vicious cycle that hampers dexterous skill acquisition. The moderate arousal levels in the current study design, apparently suppress fight-or-flight responses, enabling the trainees to start with low speeds (Fig. 4) that are appropriate for their level of expertise. This facilitates progress towards a lower error rate - thus, higher proficiency scores - (Fig. 3), locking the trainee on a positive reinforcement loop rather than a negative one.

It is worth noting that most of the previous studies focused on inanimate laparoscopic training, while the present study is centered on inanimate microsurgical training. While both forms of surgery are technically challenging^21^ and feature similar standardized tasks, they also have some differences. One key difference is the use of loupes versus endoscopic cameras for visual guidance. Hence, a question arises about the extent to which the type of surgery affects dexterous behaviors. Harwell *et al*.^22^ showed that human distress in the form of physiological tremor is inversely related to microsurgical proficiency - a finding that confirms microsurgery is no different than laparoscopy when it comes to sympathetic effects on novices^1^. In this respect, the results of our study are relevant to surgical training in general, and signal the need for a radical rethinking of educational norms and practices.

## Data Availability

The data that support the findings of this study are available at the Open Science Framework: https://osf.io/9he58/.
